# Substance abuse, treatment needs and access among female sex workers and non-sex workers in Pretoria, South Africa

**DOI:** 10.1186/1747-597X-4-11

**Published:** 2009-05-27

**Authors:** Wendee M Wechsberg, Li-Tzy Wu, William A Zule, Charles D Parry, Felicia A Browne, Winnie K Luseno, Tracy Kline, Amanda Gentry

**Affiliations:** 1 Substance Abuse Treatment Evaluations and Interventions Research Program, RTI International (Research Triangle Institute), 3040 Cornwallis Road, PO Box 12194, Research Triangle Park, North Carolina 27709-2194, USA; 2 Department of Psychiatry and Behavioral Sciences, Duke University School of Medicine, Duke Clinical Research Institute, PO Box 17969, Durham, North Carolina 27715, USA; 3 Gillings School of Global Public Health, The University of North Carolina at Chapel Hill, 135 Dauer Drive, Chapel Hill, North Carolina 27599-7400, USA; 4 Alcohol and Drug Abuse Research Unit, Medical Research Council, PO Box 19070, Tygerberg (Cape Town) 7505, South Africa; 5 Department of Psychiatry, Stellenbosch University, PO Box 19063, Tygerberg (Cape Town) 7505, South Africa

## Abstract

**Background:**

This study examined cross-sectional data collected from substance-using female sex workers (FSW) and non-sex workers (non-SW) in Pretoria, South Africa, who entered a randomized controlled trial.

**Methods:**

Women who reported alcohol use and recently engaging in sex work or unprotected sex were recruited for a randomized study. The study sample (N = 506) comprised 335 FSW and 171 female non-SW from Pretoria and surrounding areas. Self-reported data about alcohol and other drug use as well as treatment needs and access were collected from participants before they entered a brief intervention.

**Results:**

As compared with female non-SW, FSW were found to have a greater likelihood of having a past year diagnosis of alcohol or other drug abuse or dependence, having a family member with a history of alcohol or other drug abuse, having been physically abused, having used alcohol before age 18, and having a history of marijuana use. In addition, the FSW were more likely to perceive that they had alcohol or other drug problems, and that they had a need for treatment and a desire to go for treatment. Less than 20% of participants in either group had any awareness of alcohol and drug treatment programs, with only 3% of the FSW and 2% of the non-SW reporting that they tried but were unable to enter treatment in the past year.

**Conclusion:**

FSW need and want substance abuse treatment services but they often have difficulty accessing services. The study findings suggest that barriers within the South African treatment system need to be addressed to facilitate access for substance-using FSW. Ongoing research is needed to inform policy change that fosters widespread educational efforts and sustainable, accessible, woman-sensitive services to ultimately break the cycle for current and future generations of at-risk South African women.

## Background

South Africa has one of the highest levels of alcohol consumption per adult drinker in the world [1]. In 2000, estimates indicated that alcohol use contributed to 7% of disability adjusted life years lost in South Africa, ranking third out of 17 risk factors studied [2]. Among patients in specialized substance abuse treatment centers, alcohol is the primary substance of abuse reported in eight of the nine South Africa provinces, with the exception of the Western Cape where methamphetamine is the primary substance of abuse reported at treatment admission [3].

The World Health Organization's Gender, Alcohol and Culture: An International Study project [4] has increased attention on the need to study gender differences in drinking and differential responses that might be useful in addressing problems related to alcohol use. In South Africa, research has shown that females drink less alcohol (by volume) and less frequently than their male counterparts [5]. Nonetheless, estimates of alcohol use among South African females indicate that approximately 30% are alcohol drinkers [1] and roughly a third of both male and female drinkers drink at risky levels over weekends [6]. One in 10 women surveyed for the Demographic Health Survey (1998) had experienced symptoms of alcohol problems (scoring 2 or higher on the CAGE assessment) during her lifetime. Women who are poor and who lack education were significantly more likely to report lifetime alcohol problems [6]. Data suggest an increase in lifetime drinking among young, Black African males and females; and that women may use alcohol and other drugs as a way to cope with current or past life stressors [7,8]. Furthermore, many poor young South Africa women conduct sex work in order to support their families, and they report that using alcohol and other drugs helps them to solicit clients and overcome their shyness [9]. Another indicator of alcohol abuse among South African females is the extremely high prevalence of fetal alcohol syndrome among South African children in several communities [10-12].

A systematic review of FSW studies around the world reported a risk of sexually transmitted infections (STIs) and HIV, but none mentioned the risk associated with alcohol or other drug abuse [13]. Very few studies have considered the substance abuse or treatment needs of FSW [14]. No studies to date have compared the severity of substance use disorders in FSW to non-SW to adequately understand their alcohol and other drug dependence, as well as possible treatment needs. These factors reinforce the critical need to reach vulnerable women to understand the differences between them and to inform intervention and treatment that focus on individualized and gender-specific issues.

A major international review of substance abuse interventions highlighted brief treatment specifically as one intervention that is likely to be effective in reducing the burden of alcohol abuse [15]. However, despite the need for treatment, females are underrepresented in substance abuse treatment facilities, with males comprising approximately 76% to 90% of treatment center patients in all nine South African provinces [3]. Black South Africans, both male and female, are also underrepresented in treatment facilities [16,17]. Efforts to reduce treatment barriers – such as street outreach, outreach in township areas, and transportation – have not been adequately adopted by the majority of treatment facilities, despite the fact that taking these steps could potentially make treatment services more accessible to disadvantaged populations, and especially to females [16].

Female substance abusers, however, are not a homogenous group. In particular, FSW may represent a subpopulation that is particularly disadvantaged in terms of access to substance abuse treatment and other services. This population of women is also of particular relevance from a public health perspective because they are considered a core HIV transmitter group [18]. Among a study of predominately FSW in South Africa, almost 60% who used alcohol and other drugs were found to be HIV positive [19]. Despite the fact that many FSW abuse alcohol, research on this population in South Africa has tended to focus on sex risk, drug use and/or violence, rather than examining substance abuse more broadly relative to other vulnerable females [20-22].

Consequently, this study aimed to (1) examine the characteristics, age of onset, and prevalence of substance abuse disorders (within the past year), including lifetime disorders, among a group of females in Pretoria who self-identified as FSW and those who self-identified as non-SW but had unprotected sex and also use alcohol; (2) examine perceived substance use problems, the need for substance abuse treatment services, and access; and (3) determine differences between FSW and non-SW on lifetime use and current alcohol and other drug use and dependence.

## Methods

### Participants

Data for this study were obtained from a randomized controlled trial among females at high risk for HIV in Pretoria. Study participants were recruited over a 3-year period (June 2004 to June 2007) in Greater Pretoria, which includes the central business district, nearby residential areas, and surrounding townships. A variety of methods (e.g., street-based outreach, fliers, and peer advocates) were used to recruit participants from target communities and areas known for illicit drug activity and sex work, including daily rate hotels, informal settlements, weekly apartment dwellings, and shelters that were identified into sampling zones.

Eligibility criteria for the study included the following: being female, at least 18 years of age, reporting alcohol use on at least 13 of the past 90 days, reporting either trading sex for money or drugs in the previous 90 days or having unprotected sex in the previous 90 days, providing written consent to participate, and providing verifiable locator information for Gauteng Province.

Based on a quick field screener that asked the eligibility questions, females who met preliminary screening criteria in the field were referred to the project office where final eligibility was determined by repeating the screener and informed consent obtained for study participation. Appointments and transportation arrangements to the project office were made for all potential participants who met the preliminary eligibility criteria. Intake data collection began with a locator form to enable outreach staff to contact participants for subsequent assessments. Field staff then conducted urine drug screens for cocaine, cannabis, opiates, amphetamines/methamphetamine, and Ecstasy use. Breath alcohol testing was performed to determine the breath alcohol concentration at the time of the interview.

Following intake procedures, study participants were assessed by self-report at a two-part baseline interview occurring 2 to 4 days apart as well as at 3- and 6-month follow-up interviews. The consent process and additional forms and baseline interview were deemed to be too time-consuming at pretesting to be conducted in one session, and therefore they were split into two intakes. In addition, the second intake increased participation for the experimental stage of the study. Data collection was performed using paper-and-pencil interviews. Consent forms, instruments, and intervention cue cards were available in English and two local languages, Sotho and Zulu, that were translated and backtranslated by South African Medical Research Council staff. All study activities were approved by RTI International's Office of Research Protection and Ethics, and the Human Research Ethics Committee at the University of Witwatersrand in Johannesburg.

This study is based on cross-sectional analysis of 506 participants, with complete baseline data as of June 2007. Among these participants, 335 (66%) reported trading sex in the past 90 days and 171 (34%) reported having unprotected sex in the past 90 days.

### Measures

The demographic variables included age (split between 18–25 years and 26–55 years), level of education (lower than 7^th ^grade, 7^th ^to 12^th ^grade, higher than 12^th ^grade), current marital status (single; involved but not living with partner; living with partner; married, separated, divorced, or widowed), and number of children (none, one, two or more). Participants were also asked if their residence or living space had running water (yes, no) and/or electricity (yes, no). Family history of alcohol or other drug abuse was also assessed (yes, no), as well as history of being physically abused (yes, no) and/or sexually abused (yes, no). Additionally, participants were asked whether they had ever been tested for HIV (yes, no); those who reported having ever been tested were asked if they had ever been informed they were infected with HIV (yes, no).

Sex worker status was assessed with the question, "Have you traded sex for drugs, money, food, clothing, shelter or any other goods in the past 90 days?" Participants who responded yes were coded as sex workers; participants who responded no were coded as non-sex workers.

Respondents were asked a series of questions about their lifetime use and age of first use of alcohol, tobacco, marijuana by itself, Ecstasy, crack, cocaine by itself, heroin by itself, marijuana and heroin mixed, cocaine and heroin mixed, Mandrax (a sedative similar to methaqualone) by itself, Mandrax and marijuana mixed, LSD, Rohypnol, and inhalants.

Alcohol and other drug use disorders (abuse and dependence) were assessed by two separate sections, used previously in the Women's II Health CoOp, asking participants specifically whether their symptoms/problems were related to alcohol or other drug use. Assessment items were consistent with the criteria specified by the DSM-IV [23], and overall showed acceptable to excellent psychometric properties, which are presented in Table 1. The following four substance abuse criteria, as defined by the DSM-IV, were assessed: (1) recurrent substance use resulting in failure to fulfill major role obligations, (2) recurrent substance use in physically hazardous situations, (3) recurrent substance-use-related legal problems, and (4) continued substance use despite persistent or recurrent social or interpersonal problems. Lifetime abuse was defined as ever meeting one or more of these four abuse criteria. Past-year abuse was defined as meeting one or more of these criteria in the previous year.

**Table 1:** Descriptive statistics and reliability information on the alcohol and drug abuse and dependence scales

**Scale Item (When was the last time that...)**	**Mean**	**SD**	**Reliability**
***Overall Alcohol Abuse & Dependency***	34.19	8.97	0.8
***Overall Drug Abuse & Dependency***	43.77	10.81	0.9
***Alcohol Abuse***	12.23	3.34	0.7
You kept using alcohol even though you knew it was keeping you from meeting your responsibilities?	2.70	1.28	
You used alcohol where it made the situation unsafe or dangerous for you, or where you might have been forced into sex or hurt?	2.98	1.23	
Your alcohol use caused you to have problems with the law?	3.43	1.03	
You kept using alcohol even after you knew it could get you into fights or other kinds of legal trouble?	3.12	1.21	
***Alcohol Dependence***	19.42	5.89	0.8
You needed more alcohol to get the same high or found that the same amount did not get you as high as it used to?	2.69	1.33	
You had withdrawal problems from alcohol like shaking hands, throwing up, having trouble sitting still or sleeping, or that you used any alcohol to stop from being sick or avoid withdrawal problems?	2.88	1.32	
You used alcohol in larger amounts, more often, or for a longer time than you meant to?	2.55	1.32	
You were unable to cut down or stop using alcohol?	2.43	1.32	
You spent a lot of time either getting alcohol, using alcohol, or feeling the effects of alcohol (high, sick)?	2.85	1.32	
Your use of alcohol caused you to give up, reduce, or have problems at important activities?	2.99	1.28	
You kept using alcohol even after you knew it was causing or adding to medical, psychological, or emotional problems?	3.04	1.30	
***Drug Abuse***	14.03	3.15	0.8
You kept using drugs even though you knew it was keeping you from meeting your responsibilities?	3.33	1.15	
You used alcohol or drugs where it made the situation unsafe or dangerous for you, or where you might have been forced into sex or hurt?	3.42	1.09	
Your drug use caused you to have problems with the law (police)?	3.71	0.79	
You kept using drugs even after you knew it could get you into fights or other kinds of legal trouble?	3.57	0.97	
***Drug Dependence***	23.39	6.50	0.9
You needed more drugs to get the same high or found that the same amount did not get you as high as it used to?	3.36	1.15	
You had withdrawal problems from drugs like shaking hands, throwing up, having trouble sitting still or sleeping, or that you used any drugs to stop from being sick or avoid withdrawal problems?	3.34	1.19	
You used drugs in larger amounts, more often, or for a longer time than you meant to?	3.26	1.21	
You were unable to cut down or stop using drugs?	3.15	1.27	
You spent a lot of time either getting drugs, using drugs, or feeling the effects of drugs (high, sick)?	3.36	1.16	
Your use of drugs caused you to give up, reduce, or have problems at important activities?	3.47	1.06	
You kept using drugs even after you knew it was causing or adding to medical, psychological, or emotional problems?	3.45	1.08	

The following seven substance dependence criteria, as defined by the DSM-IV, were assessed: (1) tolerance, (2) withdrawal, (3) using the substance in larger amounts or over a longer period than intended, (4) persistent desire or unsuccessful attempts to cut down or stop substance use, (5) spending a great deal of time obtaining or using the substance or recovering from its effects, (6) reducing or giving up important social, occupational or recreational activities because of substance use, and (7) continued substance use despite knowledge of a physical or psychological problems. Participants who met at least three dependence criteria in their lifetime were classified as lifetime dependence. Past-year dependence was restricted to individuals who met at least three dependence criteria in the year preceding the interview. For both abuse and dependence, the same logic was applied to alcohol and other drug use diagnoses.

The subset of participants who met the criteria for past-year alcohol or other drug abuse or dependence were also assessed on perceived alcohol and other drug problems, their knowledge of treatment programs, whether they had ever called a treatment program for information or counseling, whether they had received treatment in their lifetime and in the past year, their perceived need for treatment, and whether they wanted to go to treatment.

### Statistical Analysis

Descriptive analyses were conducted on the complete sample and chi-square tests were conducted to determine the difference in demographics, socioeconomic status, history of abuse, family history of substance abuse, HIV testing and status, and substance use characteristics between the two groups of FSW and non-SW. Logistic regression models were used to identify the characteristics associated with past-year (recent or active) alcohol and other drug abuse disorders. Finally, the analysis examined whether the FSW were different from the non-SW in self-perceived substance use problems, use of substance abuse treatment, and perceived need for treatment.

## Results

There was no significant difference in age between the FSW and non-SW; however, the FSW were slightly older (Table 2). Significant differences in education and current marital status were noted, although both groups were very similar in the number of children. Large differences were found in regard to living conditions, with less than 50% of the FSW reporting electricity and running water where they live. The FSW had significantly higher rates of reported physical and sexual abuse, although high prevalence rates pertain to both groups overall. Alcohol use onset was very similar between the two groups in regard to whether drinking began before or after age 18. Significant differences were found with regard to marijuana use, with two thirds of the FSW reporting use compared with only a third of the non-SW.

**Table 2:** Sociodemographic characteristics, by sex worker status

**Characteristic, Column %**	**Overall** **N = 506**	**Female sex workers^a^** **N = 335**	**Non-Sex workers** **N = 171**	**χ^2 ^test^b^** ***p*-values**
**Age Group (years)**				
18–25	47.4	44.8	52.6	0.094
26–55	52.6	55.2	47.4	
				
**Years of Education**				
Lower than 7^th ^grade	15.4	21.5	3.5	<0.001
7^th ^to 12^th ^grade	76.9	74.3	81.9	
Higher than 12^th ^grade	7.7	4.2	14.6	
				
**Current Marital Status**				
Single, without a main sex partner	22.9	34.3	0.6	<0.001
Not living with a main sex partner	48.3	37.0	70.2	
Living with a main sex partner	24.7	26.5	21.1	
Married, separated, divorced, or widowed	4.2	2.1	8.2	
				
**Number of Children**				
None	33.8	34.3	29.8	0.186
One	33.4	31.0	39.2	
Two or more	32.8	34.6	31.0	
				
**Residence or Living Space with Running Water, Yes**	77.7	69.3	94.2	<0.001
				
**Residence or Living Space with Electricity, Yes**	59.3	43.3	90.6	<0.001
				
**Family History of Alcohol or Drug Abuse^c^, Yes**	70.2	66.0	78.4	0.004
				
**History of Being Physically Abused^d^, Yes**	53.8	63.3	35.1	<0.001
				
**History of Being Sexually Abused^e^, Yes**	30.8	37.6	17.5	<0.001
				
**Age of First Alcohol Use**				
Before 18	46.4	45.4	48.5	0.500
18 or older	53.6	54.6	51.5	
				
**Age of First Marijuana, Dagga, or Ganja Use**				
Never used	44.9	32.5	69.0	<0.001
Before 18	19.0	20.3	16.4	
18 or older	36.2	47.2	14.6	

^a ^Female sex workers are defined as females who had traded sex for drugs, money, food, or other goods in the past 90 days.

^b ^Pearson chi-square test

NS: *p *> 0.05

^c ^Family history of alcohol or other drug abuse: Ever having problems with alcohol or other drug use by any biological family member of the respondent.

^d ^History of being physically abused: Ever being physically hurt by striking or beating to the point that the respondent had bruises, cuts, or broken bones.

^e ^History of being sexually abused: Ever being pressured or forced to participate in sexual acts against the respondent's will.

Table 3 presents lifetime substance abuse overall and by both groups of females. Significant differences were found with most drugs examined, with higher rates always among the FSW. The main substances that show prevalence, aside from alcohol, are tobacco (67%), marijuana (55%), and crack cocaine (13%). Marijuana (Dagga) mixed with Thai white (i.e., heroin; 8%) and Thai white alone (7%) are the next most commonly used drugs, but use is significantly different between the two groups. There is little use of club drugs, such as Ecstasy (3.4%) and LSD (0.4%).

**Table 3:** Lifetime substance use, by sex worker status

**Prevalence of Lifetime Substance Use, Column %**	**Overall** **N = 506**	**Female sex workers^a^** **N = 335**	**Non-Sex workers** **N = 171**	**χ^2 ^test^b^** ***p*-values**
Alcohol^c^	100	100	100	.....
Tobacco	67.4	73.4	55.6	<0.001
Any drug^d^	58.1	71.9	31.0	<0.001
Marijuana/Dagga/Ganja	55.1	67.5	31.0	<0.001
Ecstasy	3.4	4.2	1.8	0.152^e^
Crack	12.8	18.8	1.2	<0.001
Cocaine	2.6	3.6	0.6	0.044
Heroin/Thai White	6.9	9.3	2.3	0.004
Dagga and Heroin/Thai White	8.1	10.4	3.5	0.007
Cocaine and Heroin/Thai White	0.6	0.9	0.0	0.554^e^
Mandrax	1.2	1.5	0.6	0.669^e^
Dagga and Mandrax	1.2	1.8	0.0	0.101^e^
LSD	0.4	0.6	0.0	0.552^e^
Rohypnol/Shabba	3.0	4.2	0.6	0.024
Inhalants	3.0	4.5	0.0	0.005
Injection drug use	0.6	0.9	0.0	0.554^e^

^a ^Female sex workers are defined as females who had traded sex for drugs, money, food, or other goods in the past 90 days.

^b ^Pearson chi-square test

NS: *p *> 0.05

^c ^Alcohol use was defined as any use of a whole alcohol drink in the lifetime.

^d ^Any drug use included the use of marijuana (Dagga or Ganja), Ecstasy, crack, cocaine, heroin (Thai White), Mandrax, LSD, Rohypnol (Shabba), or inhalants.

^e ^Fisher's exact test was used due to expected cell counts < 5.

Table 4 presents the prevalence rates of substance abuse disorders overall and between the two groups of females. Significant differences were found, with the FSW having more lifetime and past-year alcohol and other drug disorders than the non-SW. Although a high proportion of the non-SW were classified as having lifetime or past-year alcohol use disorders, significant differences were found, with higher rates among the FSW in all the categories of alcohol use diagnosis. Compared with the non-SW, the FSW also had significantly higher prevalence of lifetime and past-year drug abuse and dependence.

**Table 4:** Substance use disorders among women, by sex worker status

**Prevalence, Column %**	**Overall** **N = 506**	**Female sex workers^a^** **N = 335**	**Non-Sex workers** **N = 171**	**χ^2 ^test^b^** ***p*-values**
**Lifetime Alcohol Use Disorder**				
Abuse^c^	73.3	80.6	59.1	<0.001
Dependence	62.3	69.3	48.5	<0.001
Abuse or Dependence	80.0	85.7	69.0	<0.001
				
**Past-Year Alcohol Use Disorder**				
Abuse^c^	68.4	75.2	55.0	<0.001
Dependence	57.5	64.2	44.4	<0.001
Abuse or Dependence	76.1	81.8	64.9	<0.001
				
**Lifetime Drug Use Disorder**				
Abuse^c^	37.5	48.4	16.4	<0.001
Dependence	29.4	39.4	9.9	<0.001
Abuse or Dependence	40.9	52.2	18.7	<0.001
				
**Past-Year Drug Use Disorder**				
Abuse^c^	32.6	41.5	15.2	<0.001
Dependence	27.1	35.8	9.9	<0.001
Abuse or Dependence	37.2	47.2	17.5	<0.001
				
**Any Lifetime Alcohol or Drug Use Disorder**	81.8	88.1	69.6	<0.001
Alcohol and Drug Use Disorders	39.1	49.9	18.1	<0.001
Alcohol Use Disorder Only	40.9	35.8	50.9	
Drug Use Disorder Only	1.8	2.4	0.6	
				
**Any Past-Year Alcohol or Drug Use Disorder**	77.7	83.6	66.1	<0.001
Alcohol and Drug Use Disorders	35.6	45.4	16.4	<0.001
Alcohol Use Disorder Only	40.5	36.4	48.5	0.009
Drug Use Disorder Only	1.6	1.8	1.2	0.723^d^

^a ^Female sex workers are defined as females who had traded sex for drugs, money, food, or other goods in the past 90 days.

^b ^Pearson chi-square test.

^c^Abuse was defined as meeting DSM-IV criteria for abuse regardless of the status of dependence.

^d ^Fisher's exact test was used due to expected cell counts < 5.

In aggregate, the FSW had a higher prevalence of any past-year alcohol or drug use disorder (84% vs. 66%). Regardless of their sex-work status, most participants abused or were dependent on alcohol alone (41%) or abused or were dependent on alcohol and other drugs (36%) in the past year. The prevalence of past-year abuse or dependence on drugs only (without alcohol) was very low; less than 2% in each group.

A logistic regression model of past-year alcohol or other drug abuse disorder with both unadjusted and adjusted odds ratios is presented in Table 5. A diagnosis of past-year alcohol or other drug abuse disorder was more likely for an FSW if she had a family member with a history of alcohol or other drug abuse, if she had been physically abused, if she had used alcohol before age 18, or if she had a history of marijuana use.  

**Table 5:** Logistic regression of past-year alcohol or drug use disorder (N=506)

**Correlates of Past-Year Alcohol or Drug Use Disorder**	**Unadjusted OR (95% CI)**	**Adjusted^a ^OR (95% CI)**
**Sex Worker **(df = 1)		
Yes	2.6 (1.7–4.0)***	1.8 (1.0–3.2)*
No	1.0	1.0
		
**Age Group **(df = 1)		
18–25	1.0 (0.6–1.5)	--
26–55	1.0	
		
**Years of Education **(df = 2)		
Lower than 7^th ^grade	1.2 (0.4–3.1)	--
7^th ^to 12^th ^grade	0.8 (0.4–1.9)	
Higher than 12^th ^grade	1.0	
		
**Current Marital Status **(df = 3)		
Single, without a main sex partner	2.2 (0.8–6.2)	--
Not living with a main sex partner	1.6 (0.6–4.0)	
Living with a main sex partner	1.9 (0.7–5.1)	
Married, separated, divorced, or widowed	1.0	
		
**Number of Children **(df = 2)		
One	0.8 (0.5–1.4)	--
Two or more	0.6 (0.4–1.1)	
None	1.0	
		
**Residence or Living Space with Running Water **(df = 1)		
Yes	0.8 (0.5–1.4)	--
No	1.0	
		
**Residence or Living Space with Electricity **(df = 1)		
Yes	0.5 (0.3–0.8)**	0.9 (0.5–1.5)
No	1.0	1.0
		
**Family History of Alcohol or Drug Abuse **(df = 1)		
Yes	2.3 (1.5–3.5)***	2.5 (1.6–4.1)***
No	1.0	1.0
		
**History of Being Physically Abused **(df = 1)		
Yes	2.6 (1.7–4.1)***	1.7 (1.1–2.8)***
No	1.0	1.0
		
**History of Being Sexually Abused **(df = 1)		
Yes	3.1 (1.8–5.4)***	1.8 (1.0–3.3)^†^
No	1.0	1.0
		
**Age of First Alcohol Use **(df = 1)		
Before 18	2.3 (1.5–3.6)***	1.9 (1.1–3.0)*
18 or older	1.0	1.0
		
**Age of First Marijuana, Dagga, or Ganja Use **(df = 1)		
Before 18	4.0 (2.0–8.0)***	2.5 (1.2–5.2)*
18 or older	3.5 (2.1–5.8)***	2.4 (1.3–4.2)**
Never use	1.0	1.0

^a ^The adjusted model included variables listed in the third column.

OR = odds ratio; CI = confidence interval

^† ^*p *≤ 0.09; *: *p *≤ 0.05; ** *p *≤ 0.01; ***p ≤ 0.001.

P-values are based on the Wald chi-square statistic.

Table 6 presents the data for females who met the criteria for past-year alcohol or other drug abuse disorders. Significant differences were found between the two groups with regard to perceived alcohol and other drug use problems, with the FSW being more likely to perceive that they have alcohol and other drug problems and being more likely to perceive that they have a need for treatment. Three fourths of the FSW reported a desire for treatment.

**Table 6:** Perceived substance use problems and treatment use that met DSM-IV criteria for a past-year alcohol or other drug use disorder

**Respondents' Perceived Problems and Use of Treatment Services, Column %**	**Overall** **N = 393**	**Female sex workers^a^** **N = 280**	**Non-Sex workers** **N = 113**	**χ^2 ^test^b^** ***p*-values**
Perceived alcohol problems	41.0	45.7	29.2	0.003
Perceived drug problems	29.3	36.1	12.4	<0.001
Perceived alcohol or drug problem	55.4	63.5	35.7	<0.001
Knew of any alcohol or drug treatment program	18.6	19.3	16.8	0.529
Perceived a need for treatment	63.4	72.9	39.8	<0.001
Wanted to go to treatment	68.2	77.1	46.0	<0.001
Tried but unable to get into treatment in past year^c^	2.6	2.9	1.8	0.731^e^
Received alcohol or drug treatment in the lifetime^d^	1.8	2.5	0.0	0.200^e^

^a ^Female sex workers are defined as females who had traded sex for drugs, money, food, or other goods in the past 90 days.

^b ^Pearson chi-square test

^c ^Included having ever called a drug treatment program for information or counseling, having ever received telephone counseling from a drug treatment program, or having ever consulted a traditional healer for drug treatment.

^d ^Included the receipt of treatment from a detox, outpatient alcohol, outpatient drug, outpatient methadone, residential addiction, or jail/prison treatment program.

^e ^Fisher's exact test was used due to expected cell counts < 5.

Knowledge regarding alcohol and other drug treatment programs was limited in both the FSW and non-SW groups. This lack of awareness may partially explain why only a very small number of females in both groups reported having tried but having been unable to enter treatment in the past year; only 2% had ever been in treatment. It should be noted that the small number of females (n = 10) who reported an unsuccessful attempt to enter treatment in the past year precludes meaningful analysis of the barriers they may have encountered.

## Discussion

This study adds to the growing knowledge base about alcohol and other drug use by highlighting key differences between FSW and non-SW in a specific region of the world. Previous studies have shown that females in this region become sex workers because they do not have other employment options and often support multiple family members [9,24]. In addition, typically boys are favored in families for completing education and girls often do not complete schooling.

However, substance abuse intersects with other risks, including sexual and physical violence. Substance use also may assuage a woman's sense of embarrassment in conducting sex work and become part of an everyday ritual, which may help to explain the greater use of alcohol among FSW and their later initiation of marijuana use. In the formative stage of this study, some women mentioned how alcohol use has helped them feel assertive in talking with men to solicit clients [9]. Similarly, research into drug use and HIV risk behavior among FSW in three South African cities (one being Pretoria) found that FSW used drugs to help them get into the mood for sex work and to engage in sex acts with strangers [21].

The analysis demonstrated that there are greater differences between FSW relative to non-SW in terms of their background and their substance use and dependency. The FSW appear to be poorer and living without many of the everyday comforts, such as electricity and running water, compared with over 90% of the non-SW who have these essential amenities in their homes. The unadjusted odds ratio of having no electricity held in the logistic model as a significant independent variable. The FSW reported a significantly greater history of both physical and sexual abuse. Most females started drinking at a similar age; although those who used other drugs also started using at similar ages. In addition, there were no significant differences in age. Education, however, was significantly different, with the FSW reporting lower levels of education, which is a key underclass issue for females worldwide, as more education often means greater employability.

Whether the lack of economic opportunities in South Africa for women leads them to sex work remains speculative, but it is clear that FSW use drugs at a greater rate. Moreover, gender inequality and employment opportunities for females continue to be problematic [25]. The greater use of alcohol and marijuana – and to some degree crack and other drugs – by the FSW relative to the non-SW may be related to the nature of sex work and their subsequent need to continue to use drugs because of dependency, which in turn may put them at greater risk for further victimization, impaired sex, and HIV.

A diagnosis of lifetime dependence and abuse also showed that the FSW experienced both problems related to alcohol and other drugs. Although almost half of the FSW perceived that they had an alcohol problem and a third believed that they had a drug problem, a greater proportion perceived a need for treatment and wanted to go to treatment; however, very few ever entered treatment because they did not know of any programs.

Research on barriers to substance abuse treatment services in South Africa has shown that Black African women experience multiple barriers as FSW or non-SW [16,26]. Studies conducted among treatment centers in Gauteng Province (which includes Johannesburg and Pretoria) between 2003 and 2004 found that only 36% of centers provided woman-focused and gender-sensitive treatment programs. In general, few facilities in Gauteng provide services aimed at addressing some of the barriers – such as funding for treatment, childcare, and programs that focus on the special needs of women – that prevent women from accessing, engaging, and being retained in treatment [27].

The fact that many of the females participating in this study were not aware of treatment services but were eager to receive treatment raises questions. Thus, a logical next step would be to help these women learn about treatment, tailor treatment programs to be sensitive to women's needs, and address their comorbid conditions (e.g., sexual and physical abuse) and contextual issues (e.g., childcare). By implication, this also raises the issue of who will care for their other children and extended family if these women are not earning an income as sex workers.

## Limitations

One limitation of this study is that the sample is not a random selection but a targeted purposive sample of at-risk females in a specific geographic area in South Africa. Some females may use alcohol and other drugs to cope with violence and/or their HIV status or simply their lifestyle. The findings about alcohol and other drug use are not generalizable to all South African females or even to all FSW. However, the findings offer additional detail about substance use within the context of the similarities and differences between these two groups of females.

Another limitation is that although the study sought females who use alcohol, because the study criteria selected females who drank 13 out of the past 90 days, there is no comparison with females who do not drink. Nonetheless, interesting similarities and differences were found between FSW and non-SW, which raises important considerations when designing and implementing intervention and treatment strategies.

## Conclusion

Health service providers in this region might consider how to better reach and treat females with alcohol and other drug problems [28]. Intervention efforts should also focus on outreach strategies to continue reaching childbearing FSWs and other vulnerable females. These efforts should also address the intersecting risks that females face in South Africa because of gender inequality, as many resort to sex work because of too few or no options [25,29].

The findings of this study show that FSW need and want services, but they may be a group that is unable to access services because of what they do to support their families and because services may not be readily available or welcoming because of the stigma associated with sex work. The data show that there is a need for treatment for this population and that barriers to access need to be addressed within the South African substance abuse treatment system.

More research is needed to determine the effects of the comorbid conditions that affect these females and, in turn, study outcomes. Areas for further research suggested by this study include a greater need to understand the factors that protect females who live in difficult circumstances from becoming sex workers, such as increased education and ways to assist in accessing treatment services. Moreover, additional research will help to inform policy change that fosters widespread educational efforts as well as sustainable, accessible services that are aimed at ultimately breaking the cycle for current and future generations of vulnerable South African women.

## Competing interests

The authors declare that they have no competing interests.

## Authors' contributions

WW conceived this study, directed the research, and revised the manuscript. LW conducted the analysis. WZ contributed to the analyses and writing. CP contributed to the writing. FB contributed to the background and writing. WL conducted the literature review and wrote the methods. TK completed the psychometrics for the revision and table revisions. AG helped with the literature revisions.
